# An exploratory mixed methods study of the acceptability and effectiveness of mindfulness -based cognitive therapy for patients with active depression and anxiety in primary care

**DOI:** 10.1186/1471-244X-6-14

**Published:** 2006-04-07

**Authors:** Andy Finucane, Stewart W Mercer

**Affiliations:** 1 General Practice and Primary Care, Division of Community-based Sciences, University of Glasgow, 1 Horeselethill Road, Glasgow G12 9LX, UK

## Abstract

**Background:**

Mindfulness Based Cognitive Therapy (MBCT) is an 8-week course developed for patients with relapsing depression that integrates mindfulness meditation practices and cognitive theory. Previous studies have demonstrated that non-depressed participants with a history of relapsing depression are protected from relapse by participating in the course. This exploratory study examined the acceptability and effectiveness of MBCT for patients in primary care with active symptoms of depression and anxiety

**Methods:**

13 patients with recurrent depression or recurrent depression and anxiety were recruited to take part in the study. Semi-structured qualitative interviews were conducted three months after completing the MBCT programme. A framework approach was used to analyse the data. Beck depression inventories (BDI-II) and Beck anxiety inventories (BAI) provided quantitative data and were administered before and three months after the intervention.

**Results:**

The qualitative data indicated that mindfulness training was both acceptable and beneficial to the majority of patients. For many of the participants, being in a group was an important normalising and validating experience. However most of the group believed the course was too short and thought that some form of follow up was essential. More than half the patients continued to apply mindfulness techniques three months after the course had ended. A minority of patients continued to experience significant levels of psychological distress, particularly anxiety.

Statistically significant reductions in mean depression and anxiety scores were observed; the mean pre-course depression score was 35.7 and post-course score was 17.8 (p = 0.001). A similar reduction was noted for anxiety with a mean pre-course anxiety score of 32.0 and mean post course score of 20.5 (p = 0.039). Overall 8/11 (72%) patients showed improvements in BDI and 7/11 (63%) patients showed improvements in BAI. In general the results of the qualitative analysis agreed well with the quantitative changes in depression and anxiety reported.

**Conclusion:**

The results of this exploratory mixed methods study suggest that mindfulness based cognitive therapy may have a role to play in treating active depression and anxiety in primary care.

## Background

Mindfulness Based Cognitive Therapy (MBCT) is an innovative, empirically validated treatment program designed to prevent relapse in people who have recovered from depression [1]. Two randomised controlled trials have found that MBCT, when taught to patients in the remission phase, reduced the rate of relapsing depression, in patients with a history of 3 or more episodes of depression, by about 50% [2,3]. Kingston et al, have also recently applied MBCT to patients with active moderate severity depression in secondary care. In a controlled trial they found improvements in depression scores and reductions in rumination scores [4].

Depression and anxiety are amongst the commonest reasons for consultation in UK general practice [5,6]. The problem of relapse in depression is a significant one and for many individuals depression is a chronic relapsing condition. In a recent review on the natural history of depression Judd concludes that "unipolar depression is a chronic and life long illness, the risk of repeated episodes exceeds 80% and patients will experience an average of 4 lifetime major depressive episodes of 20 weeks duration" [7]. While a high percentage of first episodes of depression are triggered by a major life event, further episodes are less likely to have such a clear precipitant [8]. A ruminative thinking style in response to low mood appears to be a key feature in relapsing depression [9,10]. Nolen-Hoeksema defines rumination as 'behaviours and thoughts that focus one's attention on one's depressive symptoms and on the implications of those symptoms' [9]. Ruminative thinking often involves extended pondering over personal shortcomings and problematic situations and perpetuates rather than alleviates the depressed state [11].

MBCT teaches participants to recognise and let go of ruminative thinking about negative affect and instead participants are encouraged to simply remain open to what is there – to experience it fully, without aversion or attachment. Hence mindfulness training involves an attitudinal shift toward difficult experience. The heart of MBCT lies in acquainting patients with the modes of mind that often characterize mood disorders while simultaneously inviting them to develop a new relationship to these modes. Patients learn to view thoughts as events in the mind, independent of their content and emotional charge.

While the two major studies cited above [2,3] have focused on patients who have recovered from depression, it is not known whether MBCT may have a wider role to play in treating chronic mood disorders during their active phase, which is when patients tend to seek help from primary care. If acceptable and effective we can envisage a number of potential advantages to such an approach. Firstly while psychotropic medication has a role to play in treating mood disorders, it is not always effective, nor is it acceptable to many patients [12]. Secondly the group-based approach with its emphasis on the development of mindfulness skills confers a number of possible benefits over both individual and group psychotherapy. Apart from treating a greater number of patients and helping to shorten waiting lists for psychological services, the mindfulness meditation format may appeal to patients who would otherwise find talking about personal problems in group therapy too threatening. By focusing on the development of mindfulness skills and basing MBCT in primary care, MBCT may be seen by patients more along the lines of adult education rather than a mental health intervention, thus helping to de-stigmatise depression and anxiety. Finally non-specific group effects, such as validation and normalisation, are likely to play an important role in the treatment of depression and anxiety.

The aim of this exploratory pilot study was to investigate the acceptability and effectiveness of MBCT in primary care for patients with a history of relapsing depression who had current symptoms of depression or depression and anxiety. A mixed method approach was adopted, involving both quantitative data (pre and post course validated depression and anxiety measures) and qualitative data from semi-structured interviews 3 months after completion of the course.

## Methods

The following research questions were considered.

1. Is MBCT an acceptable intervention to patients with anxiety and depression?

2. What benefit, if any, do patients derive from the mindfulness approach? (Does meditation practice aggravate depression?)

3. Do patients continue to employ mindfulness techniques to cope with adverse mental states, three months after the course has finished?

4. Does an 8-week course result in improved mood as measured on Beck Depression Inventory (BDI-II) and Beck Anxiety Inventory?

### Design

Participants with a history of depression who had depression or depression and anxiety were recruited from a single practice in Ayrshire, Scotland. Two researchers were involved in the study; AF a General practitioner (GP) with training in CBT, meditation and MBCT and SWM, a GP and senior clinical research fellow at the University of Glasgow, with training in MBCT and experience in qualitative research. AF had previously completed the MBCT 8-week course as a participant to gain first hand experience of the process. Further training was undertaken in the form of an intensive course with the North Wales Centre for Mindfulness Research and Practice (University of Wales) to become an MBCT instructor. Only AF was involved in the delivery of the MBCT program and SW conducted the interviews.

### Ethical issues

Ethics approval was obtained from Ayrshire and Arran local ethics committee and informed consent was obtained from all participants prior to inclusion in the study.

### Recruitment

Information governing selection criteria was distributed to participating GPs (table 1). GPs gave patients who met these criteria an information leaflet about the MBCT study. Those who expressed an interest in taking part were assessed prior to enrolment. The assessment process took 1–1.5 hours and involved a detailed history in order to establish a provisional ICD-10 diagnosis (table 2).

**Table 1:** Inclusion and exclusion criteria

***Inclusion criteria***
• Patients aged 18–65 with history of Recurrent Depressive Disorder or Depression and Anxiety.
• Current symptoms of depression lasting > 2 weeks (see table 2 for ICD-10 criteria).
• At least 2 episodes should have lasted a minimum of 2 weeks and should have been separated by several months without significant mood disturbance (i.e. its recurrent).
• Have BDI score of more than 14 (i.e. mild depressive disorder or worse – case definition).
• With or without primary symptoms of anxiety.
***Exclusion criteria***
• Patients with organic brain disease.
• Currently know to be abusing drugs or alcohol.
• History of psychosis or mania.
• Diagnosed personality disorder.
• Currently suicidal (score of 2 or more on the BDI-II suicidal thoughts item).
• Unable to participate in MBCT program (as measured by a score of 7 or more on the combined BDI-II items for energy, concentration difficulty and Tiredness).

**Table 2:** ICD 10 definition of depression

**Typical **symptoms are
• Depressed mood – varies little from day to day, often unresponsive to circumstances. May show diurnal variation. Can be masked by added features such as irritability, alcohol, and histrionic behaviour.
• Loss of interest and enjoyment.
• Reduced energy leading to increased fatigability and diminished activity.
**Other **symptoms are
a) reduced concentration and attention
b) reduced self-esteem and self-confidence
c) ideas of guilt and unworthiness
d) bleak and pessimistic views of the future
e) ideas of self harm
f) disturbed sleep
g) diminished appetite
**Mild Depressive Episode: **Two 'typical' symptoms plus at least 2 'other' for 2 weeks. Usually distressed by the symptoms and has some difficulty in continuing with ordinary work and social activities, but will probably not cease to function completely.
**Moderate Depressive Episode: **Two 'typical' plus at leas 3 'other' preferable 4 and to marked degree.
**Severe Depressive Episode: **Considerable distress or agitation (or retardation).

### Participants

In total 16 patients were offered pre course interviews. Three people were excluded from the study, leaving 13 to participate; one because her symptoms were attributable to sleep apnoea syndrome, one because he was actively suicidal and one because she scored greater than 7 or more on the combined BDI-II items for energy, concentration difficulty and tiredness.

### Intervention : The MBCT course

The structure and format of the mindfulness course closely followed that of the original 8-week MBCT course, found in Williams, Segal and Teasdale's book "*Mindfulness Based Cognitive Therapy; a new approach for relapsing depression" *(2000). This course teaches a variety of methods for developing mindfulness:

a) The body-scan – becoming aware of bodily sensations.

b) Guided Sitting meditation – cultivating a decentred awareness in relation to physical sensations, sound and cognition.

c) Mindful Stretching and Mindful Walking – developing awareness of bodily sensations through movement

d) The 3-minute breathing space – an exercise in bringing attention into the present moment, developing a greater awareness of the effects of difficult experience on thoughts, feeling and physical sensations.

e) Mindfulness in everyday life – bringing awareness to routine tasks such as eating and washing

Because concentration is affected in depression, a decision was taken to shorten some of the longer meditations; the body scan was reduced from 40 to 30 minutes and the guided sitting meditation reduced from 40 to 25 minutes. Shortening the practices is a contentious issue within MBCT circles. While on the one hand mindfulness training involves developing a more decentred approach to difficult experience, and longer meditation sessions provide a greater opportunity to encounter experiences such as frustration, physical discomfort and painful emotional states, this in turn must be balanced by participants ability to be able to 'stay with' these difficult experiences. Because the participants in this study had a range of affective symptoms we believed that shorter sessions with these patients were as likely to produce difficulties as longer meditation sessions in recovered depressed patients.

### Interviews

SM, an experienced interviewer and qualitative researcher, conducted one-to-one semi-structured interviews, with 11 out of the 13 participants at a mutual agreed location. Some of the interviews were done face to face and others were done by telephone. The interviews, which followed a relaxed conversational style and covered issues indicated in (table 3), lasted approximately 30–45 minutes. All were recorded and transcribed verbatim. After the first few interviews were conducted the interviewer added other questions based on themes that had emerged from the previous interviews. For example specific questions were asked about 'being in a group' or 'impressions of the facilitator'.

**Table 3:** Qualitative Interview format

Semi-structured using questions around 5 themes
1. Participants overall impressions – "in general what did you think of the overall approach?"
2. The course techniques/methods/materials – "what aspects of the course did you find beneficial", "what aspects of the course did you find difficult/unhelpful?"
3. The format of the course – "what did you think about the length of the course?"
4. Ongoing mindfulness practice – "are there any techniques you continue to use?"
5. Coping skills – "do you feel better able to cope with adversity than before you started the course?", "has anything changed for you since you completed the course?"

Two participants were not interviewed; one because she had moved out of the area following a break down of her marriage and the second participant because she could not be contacted. All the other participants agreed to be interviewed.

### Type of analysis

Both quantitative and qualitative methods were used. The Beck depression and anxiety inventories provided numerical data and the qualitative component elaborated on the numerical relationships, helping to make sense of the numbers.

### Qualitative analysis process

The interviews were transcribed verbatim and the audio interviews checked against the transcripts to ensure accuracy. Both researches reviewed the data independently and a set of preliminary concepts or codes was generated. Using the qualitative software package *Nvivo*, the transcripts were coded using these preliminary concepts or new concepts as they arose. The process was continuous and iterative and from this a number of major and minor themes emerged. Using a framework approach [13] the data was synthesised into a smaller number of thematic matrices. Each thematic matrix contained data from the interviews pertaining to the theme in question, alongside participant information such as demographics, number of sessions attended, baseline symptoms and BDI-II/BAI scores. This provided a wider context in which to view the data.

Because of the subjective nature of qualitative research and the potential for researcher bias, the analysis remained predominantly descriptive rather than interpretive, allowing patients' narratives speak for themselves. Particular attention was directed not only at emergent themes that were similar to each other but also to looking at data that diverged from the norm.

## Results

### Participants

Table 4 shows the profile of the individual participants. Three participants were male and ten female. The average age of the group was 43 (range 29–58). The average pre-course BDI score was 36.0 (range 23–46) and pre-course BAI score 32.0 (range 10–49). A breakdown of ICD-10 diagnoses was as follows.

**Table 4:** Baseline characteristics for participants

**Age**	**Demographics**	**BDI** **(0–63)**	**BAI** **(0–63)**	**Diagnosis and previous treatment**
50	Married Male. Self employed NS-SEC 4	38	35	Current: Generalised anxiety disorder. Recurrent depressive disorder – moderate. Past : Post traumatic stress disorder. Previous treatment : CBT 5 year – 8 sessions specific for PTSD. Medication : cipramil 40 mg (5 years).
38	Married housewife. NS-SEC 8	26	43	Current : Generalised anxiety disorder. Recurrent depressive disorder – moderate. Repeated in patient hospital admissions for severe anxiety/agitation. Medication : Clomipramine 50 mg.
58	Divorced female NS-SEC 8	42	46	Current : Recurrent Depression current episode moderate with severe anxiety. Recently discharged from psychiatric hospital. Past – phobic anxiety disorder and depression. Previous ECT. Medication : Venlafaxine 75 mg BD and propranolol.
29	Single female NS-SEC 8	46	27	Current: Recurrent Depressive disorder, current episode moderate. Past : post natal depression. Medication : Zopiclone PRN. Previously on cipramil.
39	female, divorced NS-SEC 7	31	25	Current : Recurrent Depressive disorder, current episode mild to moderate. Medication : None. Previously on cipramil *3 years.
56	Married Male. NS-SEC 3	44	27	Current : Recurrent depressive disorder current episode moderate. Medication : Cipramil 40 mg (6 years)
36	Single male NS-SEC 3	23	20	Current : Mixed anxiety and depressive disorder. Past : Recurrent depressive disorder. Medication : Stopped venlafaxine one month prior to course.
58	Divorced female NS-SEC 8	30	10	Current : Recurrent depressive disorder, current episode mild to moderate. Past : Severe depression and GAD. Saw psychologist 10 years ago after 'nervous breakdown'. Medication : cipramil 20 mg (2 years)
40	Married female NS-SEC 3	42	49	Current : Generalised anxiety disorder. Major depressive disorder current episode mild to moderate. Medication – cipramil 40 mg
35	Single female NS-SEC 7	44	42	Current : Recurrent depression current episode moderate. Medication – propranolol for anxiety. Previously on antidepressant for 2 years.
42	Divorced female NS-SEC 3	27	26	Current : mixed anxiety and depression. Past – recurrent depressive disorder.
41	Divorced female NS-SEC 8	37	34	Current : Major depressive disorder – moderate. Moderate anxiety. Past : alcohol abuse. Social phobia. ? PTSD. Health anxiety – treated with CBT 10 years ago. Medication – diazepam PRN.
38	Married Female NS-SEC 8	38	32	Current : Recurrent Major Depressive disorder – current episode moderate. Past : PTSD Currently on amitriptyline and weak opiod analgesia for pain.

*NS-SEC National Statistics Social-Economic Classification (NS-SEC)

NS-SEC = National Statistics Social Economic Classification

This is a widely used socio-economic indicator based on classification of occupation as follows;

NS-SEC 1: Higher managerial and professional occupations

NS-SEC 2: Lower managerial and professional occupations

NS-SEC 3: Intermediate occupations

NS-SEC 4: Small employers and own account workers

NS-SEC 5: Lower supervisory and technical occupations

NS-SEC 6: Semi-routine occupations

NS-SEC 7: Routine occupations

NS-SEC 8: Never worked and long-term unemployed

• 11 participants had an ICD diagnosable depression, 9 of whom satisfied ICD-10 criteria for *recurrent depressive disorder – current episode mild/moderate or severe*. The other 2 participants with depression had primary diagnosis of *generalised anxiety disorder*.

• 2 participants had *mixed anxiety and depressive disorder *and past histories of depression.

Two participants also had a past diagnosis of post-traumatic stress disorder given by a consultant psychiatrist. Two others probably had past diagnosis of PTSD and two individuals also divulged a history of significant childhood sexual abuse. Overall there was a significant degree of psychological morbidity in the group.

### Qualitative results

#### Preconceptions, motivations and expectations

While the majority of participants had past experience of some form of psychological intervention (10/13), ranging from counselling to relaxation exercises, and five had seen a psychiatrist, only one participant had past experience of meditation. Several people in the group indicated that the chronicity of their problems with anxiety and depression was a major motivating factor for participating in the program.

"I was eh, at a stage, I still am at a stage, where I will do anything"-P1

"Because I have suffered for a lot of years with anxiety and bouts of depression"-P9

Avoiding medication was highlighted by only one of the participants as an important reason for persisting with the course.

"I didn't want anti-depressants or things like that. I wanted to take control of things myself... ...I was determined when I started to stick to it, to get to the end of the course and understand what the course was about. I also went into it thinking this might not help me, but I am giving it a try. I am going to see it through and it did help."-P5

A few participants mentioned the importance of commitment and self-help as an important part of recovery from depression.

" I've always had the attitude that if I don't do it nobody else can do it for me. So you know, it was there and I wanted to try it and I got the opportunity to do it. So it was a sort of self help thing"-P9

"You have got to work at these things you know... ...my downfall is I'm very undisciplined I don't follow things through"-P8

"(the MBCT course was) Something I could work with and something I saw myself practising out"-P6

One woman, who had struggled with severe anxiety and depression for 30 years, described having high expectations prior to starting the course and feeling disappointed afterwards.

"*Because I've got severe anxiety and depression so I thought coming along to this I would be immediately cured – so I am disappointed in that"-P3*

#### Being in a group

This was the first time that any of the participants, except one, had been in a group 'intervention'. For several people this seemed to be an important normalising process. Themes such as being understood by the group, realising that you were not alone and being able to show emotion in a safe environment, emerged as common positive aspects to being in a group.

"It was really good and I got to know other people as well, that I'm not on my own. There is other people with the same sort of problems which is good"-P10

For one man being in a group was an important turning point in his understanding of mental illness;

"Don't ask me, what I was expecting the other people to be? Raving lunatics, people with axes in their hands, I haven't a clue – but they were not....it was you, it was my next door neighbour. They weren't giggling half wits. I know that is rather narrow minded but they were ordinary everyday run of the mill people which reinforces the fact that that is what I am as well. I'm not a nut...I'm just an ordinary, everyday run of the mill person who ended up in the crap for whatever reason, and so are they. So that was another thing that was a great plus"-P1

However, not everyone found being in a group a positive experience. One man with a history of panic attacks, usually provoked in social circumstance, found the group claustrophobic. For him, the group conjured up images of an Alcoholics Anonymous meeting and he was afraid that he might become more depressed if he stayed in the group;

"I found I was a lot better than I had been when I started the course anyway, with going back to work. And things seemed to be picking up. But I found, I wouldn't say it dragged me back down, but I felt it started to almost like reawaken kind of feelings of anxiety being in close proximity to erm, so many people and just in the group that kind of thing you know"-P7

Two people talked about how the group helped them persevere with the meditation exercises.

"I think if you are on your own you would quite easily walk way and give up whereas you've got the support there and you know that everybody's sort of helping you out and you would go back in for the groups sake and try again."-P9

Several people in the group expressed relief about not having to talk about their personal problems.

"I didn't like it at first...in case I have to say what my problems were....You were under no pressure whatsoever. If you wanted to come in and not participate that night, just sit, watch and listen that was fine by him"-P6

#### Length of the course

While most of the group found the course enjoyable the majority of the group thought the course was too short. Although some participants commented that it was long enough to learn the basics of practice, most of the group would have liked the course to go on for another 3–4 weeks. Nine out the eleven interviewed expressed a desire for some form of follow-up and one woman spoke about feels of loss of support following completion of the course.

"I felt it was about right....... what a few of us was saying, actually at the end we wished it would continue for a wee bit longer cause we enjoyed it so much ... ...the people I was getting up to the bus stop at night that's what they where saying, they would have loved it to have continued."-P5

"I think it could have been longer, much longer... ...maybe another 4 weeks on top of that would have been better."-P11

"If we even said ...monthly or quarterly something like that whereby you have still got this link and you would still have each other. It's like you had this sort of support if you like and then it's just gone. So I think personally a follow up is a must"-P9

"I know meditation would be a good thing and I would enjoy it if I could get into it, it's very beneficial and I think it would have helped if it had gone on longer but it just wasn't long enough."-P3

One participant thought that some additional one-to-one sessions with the course facilitator would allow participants discuss personal problems without having to air them in front of the group.

#### The course exercises

There was a wide range of views on the course exercises, in particular the body-scan and walking meditation. While some participants found the body-scan a pleasurable, relaxing experience others found it a difficult practice.

"I liked the body-scan. That was the one bit that I really liked. I've got an awful lot of pain with the arthritis and when he was going through the body-scan and all that saying breathe into the pain, it was actually taking the pain away."-P3

"What I did find about the body scan when we were doing it whether at home or in here I became very aware of small itches and things like that irritated by them and really that was part of what I found quite hard to do. If it was my foot throbbing or itchy or just if I start to feel I've got to get out you know I've got to get up"-P7

One woman with a history of childhood sexual abuse found the body-scan made her aware of "*horrible feelings through my body that I had never felt before*". She found this exercise and the longer meditation exercises too difficult to practice at home. Despite this, she found the 3-minute breathing space a useful exercise and continued to use it regularly, three months after the course had finished. In contrast, one of the other participants with a history of childhood sexual abuse had no such problems with the body-scan and continued to practice the longer meditation practices several times a week three months after the course finished. One man had a traumatic flashback of an accident he had witnessed while doing the body-scan for the first time. This flashback, the first for a number of months, provoked considerable anxiety and claustrophobia. Another participant with severe generalised anxiety disorder found the body-scan an effective way of reducing her anxiety and found it more effective than her previous experiences using a progressive muscle relaxation technique.

*"I couldn't believe the way I was feeling after doing the body-scan.....when you are doing (progressive muscle) relaxation you are sort of concentrating just on muscles or different parts of your body but it's outside your body but I felt the meditation was going inside the body.... as if I've got into the root, is probably the best way to describe it. And I can get right to the nucleus of it and I can feel it"-P9*.

There was a wide variation in the amount and type of homework done, with some participants only practicing the occasional breathing space and other spending 30 minutes a day practicing meditation. One woman felt that making the time to practice the longer meditation was '*too much of a luxury' *when she had 6 children at home an instead practiced mindfulness of washing the dishes and mindful walking.

*"you are doing the dishes.... actually take the time, look at the shape, the shape of the dishes and the water temperature. Calm down and actually take notice of what you are doing and relaxing instead of automatically jumping, as I do, onto the next couple of things maybe for the whole day, you know*.-*P2*.

Some participants described struggling with the meditation exercises;

"I have great difficulty in keeping on the line of the meditation, getting used to it, I had great difficulty but I still persevered and I still have great difficulty with it"-P1

"The one where you meditate sitting, I can't do it for 25–30 minutes because I've got a bad spine and I found it, I get agitated I cant sit for that length of time"-P8

Others adopted a more flexible attitude towards practice;

"Sometimes I would start doing it (the body-scan) and maybe I didn't feel myself relax. I fought against it at the start. I thought this isn't working but what I started doing was if I didn't feel that I could relax right away I would put it off and then later on go back and do it".-P5

The same participant later she describes letting go of trying to force the relaxation

"I let go of those feelings and it just all started to come naturally."-P5

Another woman described a similar process whereby her ability to sit with her anxiety depended on her own meditation skill and the degree of anxiety;

"I find it (sitting meditation) very, very good but I must say that when I am very, very anxious and uptight about something I find it very hard, very, very hard to sit with my anxiety.... that's a definitely a skill".-P11

In general, those in the group that were able to let go of expectations of results and focused simply on the meditation methods, were more likely to persist with the exercises and feel benefit from the course.

#### Benefits and on-going practice

Most of the course participants continued to use some of the mindfulness exercises three months after the course ended, suggesting that they found some benefit from these practices. The majority continued to use the three-minute breathing space, finding it an effective method for regaining composure in the face of difficult emotions, particularly anxiety. Five participants continued to have a regular formal meditation practice 2–3 times per week three months after finishing the course. Other participants, while not continuing formal periods of meditation, integrated mindfulness practices into ordinary activities such as walking the dog or washing the dishes. Several participants spoke about the difficulty in motivating themselves to continue practicing, after the course had ended.

In total 4 participants dropped out of the course and two of these were interviewed. One man had kept the material with the intention of one day trying it. The other, who dropped out after the first session said the course had acted as a trigger for her to engage in her own form of meditation/relaxation practice. Of the two drop outs that were unavailable for interview one had a history of alcohol abuse and dropped out after only 2 classes. The other, a woman whose husband had walked out on her half way through the 8-week program was too upset to continue with the course, had since moved out of the area.

The group described a wide range of benefits that came from the course. These included an

• increased ability to relax,

• a decreased tendency to jump to negative conclusions,

• learning to take time out,

• learning new ways of dealing with difficult emotions

• greater self acceptance.

*"I am able to deal with my emotions...I am not scared of things any more...I don't want to turn about and walk away from things...I'll take the time out to sit down and face up to it..."-*P5

"Well I think it must have helped because I usually land up in hospital and I didn't this time... ... I'm just being more relaxed about what I am thinking"-P2

"I don't panic the same, eh, I still have negative thoughts about things, I worry a lot and I always see the pessimistic point of view but I don't go into tizzies.... the course has helped. I wish I had that course years ago"-P8

"its helped me look at things in a different way...just accept it"-P3

Two participants who had been off work because of psychological difficulties believed the course had helped them get back to work. One of these participants had been out of work for almost a year because of depression and difficulty coping with stress at work. He felt the course helped him get back to work. The other participant had been off work for 9 months due to a combination of physical and psychological problems.

"I do the 3 minute thing when I'm at work.....and to be honest with you I feel that if I didn't do it I would have to go home, you know, I would have to leave my work"-P9

One woman who found the course especially useful, and whose depression resolved completely, described how she had discovered self-worth and joy.

"*I feel more worthwhile now. I'm beginning to feel now that there is something out there for me. I'm going to go back to work as well.......My outlook has changed. The kids have even noticed it"-P5*

*"I had tried anti-depressants and that and I'm not really one for taking medication if I can help it and I think something like this, it doesn't make you be in control of your life, but it certainly helps and I think that is the thing, if something can help you. Whereas the anti-depressants I just felt as if I wasn't in control anymore. They made me feel different. The same problems were there. So when I stop taking the tablets I still had the emotional baggage and everything that I had stopped feeling when I started taking the pills. It was waiting for me at the end of the course whereas I feel with this, this is a different course. I've dealt with everything myself and at the end of the course they feelings are still there but I can deal with them so I would definitely feel that this is an alternative"-P5*.

For one woman with generalised anxiety disorder the course gave her a method of managing her anxiety when having a medical procedure;

*"I got a lot out of the body-scan. There was an incidence where I had went to the hospital for an endoscopy and you hear all the horror stories about what is going to happen and whatever and normally with things like that I would be physically shaken, you know I would be so uptight but because I had this, under my belt if you like, I thought no I've got to use it, that is what it is there for, so I did use it and I wasn't shaken and I was so proud of myself"-P9*.

Several participants found their sleep improved when they practiced mindfulness meditation and one man found that mindfulness meditation techniques helped him with cope with restless legs syndrome. One woman describes how techniques learned for dealing with anxiety helped her give up smoking.

"But this time I stopped smoking ...and I have still stopped and I'm sure that course helped me.... I don't know if you know anything about the patches, the last month we go on a low dose, it really is quite hard then because you are coming off the nicotine and I get really, really anxious. And I really do think if it hadn't been for that meditation that 8 weeks I maybe would have started smoking again"-P11

Learning to live in the present moment was seen as a way of letting go of anxiety and re-discovering joy. One woman, saw the course in spiritual terms:

"Because what its (meditation) actually accentuating is the five senses ... taking in what your seeing, what your hearing, to when your eating something; you notice the texture. I walk my son's dog and I really had a lovely calming experience. It was a lovely day and I was watching the lovely breeze in the trees and I was watching the flowers and the river and your really more conscious of creation so I felt that the spiritual connotations were what was different,-P8

While some members of the group described very positive changes in mood and attitude as a result of completing the 8-week course, other participants found the course less helpful. One participant, who had suffered from anxiety and depression for more than 30 years, had hoped meditation would provide a 'miracle cure' and was disappointed this had not been the case. She spoke about on going family problems, expressing feelings of rejection and isolation and continued to experience high levels of anxiety. While she enjoyed the classes and found the 'thoughts and feelings' exercise very informative, at times she felt overwhelmed by the amount of new information. Importantly, she tended to conceptualise mindfulness practice in terms of relaxation alone and remained goal oriented while practicing meditation. Because of this she found herself judging her practice as successful if it induced relaxation and unsuccessful if she was tense or distracted. During the classes she found herself able to relax, but at home she spoke about becoming easily distracted by noises which she felt interfered with her practice and so in her opinion made the practices less effective.

"Nothing has got worse. Just I know meditation would be a good thing and I would enjoy it if I could get into it. It's very beneficial and I think I it would have helped if it had gone on longer but it just wasn't long enough. I have still been putting the tape on but I'm not putting into it, you know, it doesn't seem to be working, I think it's because I know I'm not coming back to the class"-P3

Another participant with posttraumatic depression and anxiety, found the course interesting but not particularly useful to him. While he found attending the group a hugely normalising experience, he found the meditation practices irritating and difficult. He spoke about becoming irritated with the audio instructions on the CD and giving up on the guided meditations early on in the course. Occasionally he meditated on sound, which he found calming, but admitted that since the course had ended this practice was diminishing. He continued to struggle with difficult emotional states and believed that while the mindfulness approach was helpful, it was only helpful to a certain degree:

"*How much that degree is I couldn't quite fathom at the moment, it's not been long enough. There are so many hurdles that you've got to jump over. It's so easy to trip up, so unbelievably easy to get yourself back into the rut....I think perhaps it makes you recognise that you are on the edge of the rut quicker rather than falling into it and saying how the hell did I get here. And it gives you some methods of holding a better balance."*-P1

### Quantitative results

Pre and post course Beck depression and anxiety questionnaires were available for 11 individuals (table 5). The mean pre-course depression score was 35.7 and post course was 17.8, with a mean before-after difference of 17.9. (95% C.I. 9.38 : 26.4). A similar reduction is noted in anxiety with a mean pre-course anxiety score of 32.0 and mean post course of 20.5. The mean reduction in anxiety was 11.45 (95% C.I 0.69 : 22.22). Effect sizes for the intervention of 1.5 and 0.77 were calculated for depression and anxiety respectively using Cohen's D statistics [21]. Cohen defined effect sizes above 0.8 as large.

**Table 5:** Mean, Median and confidence intervals for Beck Depression and Anxiety inventories before and 3 months after MBCT.

	Depression	Depression	Anxiety	Anxiety
	Pre	Post	Pre	Post

Mean	35.73	17.82	32.00	20.54
Lower bound 95% C.I. for mean	29.88	8.02	23.59	9.07
Upper bound 95% C.I for mean	41.57	27.61	40.40	32.01
Median	38.00	11.00	27.00	18.00
Std. Deviation	8.69	14.59	12.50	17.08
Min	23.00	0.00	10.00	1.00
Max	47.00	42.00	49.00	58.00
Interquartile range	17.00	25.00	21.00	23.00
Skewness	-0.220	0.404	-0.115	1.03

## Discussion

In the present study four research questions were considered;

1. Is MBCT an acceptable intervention to patients with depression and anxiety?

2. What benefit, if any, do patients derive from the mindfulness approach?

3. Do patients continue to employ mindfulness techniques to cope with adverse mental states, three months after the course has finished?

4. Does an 8-week course result in improved mood as measured on Beck Depression Inventory (BDI-II) and Beck Anxiety Inventory

### Is MBCT an acceptable intervention to patients with depression and anxiety?

The majority of the participants found the MBCT course acceptable, enjoyable and beneficial. However most of the group also felt the course was too short and thought that some form of follow up was essential. For many of the participants, being in a group was an important normalising and validating experience. Their description of the facilitator as an empathic listener who taught from his own experience contradicts the notion that mindfulness training is a detached therapy.

Duration and severity of illness, avoidance of medication and desire to engage in a form of self-help, were cited as factors that motivated participants to complete the course. Interestingly two of the three patients who did not complete four sessions had relatively mild mental health histories compared with the rest of the group. This is in keeping with previous findings that found a significant increase in drop out rates for those with two episodes of depression compared with three or more [3]. This would support the hypothesis that duration of illness is an important motivating factor for engaging with mindfulness based cognitive therapy.

### What benefit, if any, do patients derive from the mindfulness approach?

Analysis of the interviews suggests a correlation between the amount of effort participants invested in developing their own mindfulness practice and improvements in psychological well-being. This is in keeping with previous findings that suggest strong links between consistent practice (therapy 'homework') and the process of change [14]. The reported benefits of mindfulness training in this present study included an increased ability to relax, improved mood, greater self-awareness and self-worth, improved sleep and new ways of working with negative thoughts and emotions. Two participants who went back to work and one woman who gave up smoking attributed these changes to skills they had developed as a result of partaking in the group.

Several factors appeared to influence participants' commitment to mindfulness training including initial experiences of mindfulness, time pressures, individual characteristics and on-going personal and interpersonal difficulties. Two members of the group who had difficult initial experiences with the body-scan, one with a history of post-traumatic stress disorder, the other with childhood sexual abuse, did less formal meditation practice during the course than the rest of the group and gave up on the longer meditation practices once the course had ended. Positive initial experiences could also be an obstacle to mindfulness practice if they created expectations that were not subsequently fulfilled: one woman for example, became frustrated and demoralised because she could not achieve the degree of relaxation she initially experienced while doing the exercises in the group. Conversely, participants who found the initial exercises relatively straightforward and who were able to adopt a relaxed, non-striving, non-judgemental approach to mindfulness practice tended to enjoy the exercises and persist with them after completing the course.

### Do patients continue to employ mindfulness techniques to cope with adverse mental states, three months after the course has finished?

Three months after the course had ended the majority (8/11) of the participants continued to use mindfulness techniques such as the breathing space. Five participants who completed the MBCT course continued to do some formal mindfulness meditation practice 2–3 times per week. Finding the time to practice and lack of group support were highlighted as obstacles to on-going practice. The lack of ongoing support was a common theme, and we would suggest that the establishment of ongoing support groups may be a very important part of long-term effectiveness.

### Does an 8-week course result in improved mood as measured on Beck Depression Inventory (BDI-II) and Beck Anxiety Inventory?

The mean pre-course depression score was 35.7 and post course was 17.8. A similar reduction was noted for anxiety with a mean pre-course anxiety score of 32.0 and mean post course of 20.5. Overall 8/11 patients (72%) showed improvements in BDI and 7/11 (63%) patients showed improvements in BAI with pre/post effect sizes of 1.5 for reduction in depression and 0.77 for anxiety. While the numbers involved in this study are small, the results compare favourably with results published using other group psychotherapies. In one uncontrolled study using a 12-week group CBT programme for the combined treatment of depression and anxiety, the authors report that 69% of the patients had an improvement in BDI and 62% an improvement in BAI [15]. Pre/post effect sizes for depression and anxiety in this study were 0.9 and 0.44 respectively.

Although post-course depression and anxiety scores (BDI and BAI) showed statistically significant improvements in mood and the majority of the participants believed they had benefited from the course, it is worth highlighting that 5 of the 11 patients continued to have significant levels of depression and or anxiety three months later (moderate to severe range). Two of these five patients reported that they found the course beneficial despite no improvement in BDI and a worsening post-course BAI. The other three patients' BDI or BAI scores moved from the severe range to moderate range. These findings are consistent with results from a meta-analysis on the efficacy of group psychotherapy for depression, which concluded – "while treated participants improved substantially participants still had pronounced depressive symptomatology relative to normative levels of depressive symptoms seen in non-depressed controls" [16].

In general there was relatively good agreement between quantitative results and the findings from the qualitative interviews with patients who reported an improvement in their affect at interview also recording positive change in their BDI/BAI scores. However, there were exceptions to this. One woman who reported feeling better than before scored higher on both BDI and BAI following the course and another participants who scores fell significantly following the course was not sure if he felt much improvement in his mood. At the present time we are unable to explain these discrepancies between the qualitative and quantitative findings.

### Interpreting the findings

There are several possible interpretations of the findings in this exploratory study, all of which require further research to answer. The acquisition of 'mindfulness skills' as a result of MBCT training may be central to alleviating affective disorders in this patient group. However it may be that these skills were not sufficiently developed and integrated into the lives of the minority of participants who did not report benefit; with additional time and practice, mindfulness training may perhaps have proven beneficial. In addition, as we have pointed out in the methods section, we took a decision to slightly shorten the body scan and guided meditation practices. It could also be that by shortening these required practices, we prevented some participants from getting 'full exposure' to MBCT training within the eight-week course. Clearly this is a contentious issue, which requires further elucidation.

On the other hand, there could be important therapeutic elements missing from the MBCT program that significantly hinder its effectiveness as a intervention for long standing depression and anxiety for certain individuals. Elements known to be effective in the treatment of affective disorders include goal setting and behavioural activation, techniques to modify unhelpful assumptions, assertiveness training, and schema work [17]. From this perspective MBCT can be viewed as one component of a larger program of treatment. A third possibility is that the improvements in mood found in the present study were not specifically related to the MBCT course itself but were due to a 'regression towards the mean' phenomenon or/and non -specific group effects. This interpretation however is at odds with theoretical and empirical support for mindfulness training [2-4,11,18].

In the present study, participants who described improvements in mood not only continued to practice mindfulness meditation on a regular basis, but also made changes in other areas of their lives. Behavioural changes included going back to work, giving up smoking and increasing exercise. Cognitive changes included recognising and disengaging from worrisome, unhelpful and self-critical thinking. Participants whose depression and anxiety resolved also had more social support and fewer ongoing interpersonal problems. Individuals with significant post-course depression symptoms tended to have decreased levels of activity (one woman continued to spend much of her day in bed), were more socially isolated and had significant histories of unresolved trauma such as childhood sexual abuse, early emotional neglect and domestic violence.

Although participants continued to use the breathing space to cope with anxiety, there appeared to be a marked difference in the application of this technique. Two of the participants used the breathing space to recognise, welcome and disengage from worrisome thinking. This is in contrast to some other members of the group who continued to engage in active worry and use the breathing space as a way to cope with physiological symptoms of anxiety. This suggests that for these patients mindfulness training did not change the way they related to unhelpful beliefs about worry ("worry is protective"/"worry will help me cope"/"worry is not controllable") known to play a significant role in maintaining generalised anxiety disorder [19]. Unlike CBT, the mindfulness-based approach does not explicitly aim to challenge beliefs about worry and instead focuses on changing participants' way of relating to worry itself. In theory, the repeated application of non-judgemental attention to the process of worry (watching thoughts come and go without either blocking or following them) reduces habitual worrying and the distress associated with worrisome thoughts. However this is a rather subtle process and as this study shows is the easily open to misinterpretation.

Although it is possible that the course was simply too short, and indeed the majority of participants thought that this was the case, the above examples indicate that for some patients MBCT may not affect significant change in key domains that maintain depression and anxiety. Interestingly, while Baer's review [18] suggest that mindfulness training has benefits across a wide range of disorders, Teasdale et al remain cautious about attempts "to apply mindfulness training indiscriminately, as if it were a simple, general-purpose therapeutic technology" [20]. Instead they suggest that mindfulness training "is best conducted by practitioners who have adequately formulated views of the disorders they wish to treat and of the ways that mindfulness training can be helpful to clients with those disorders". In the case of relapsing depression the problem is conceptualised in terms of ruminative thinking induced by low affect, which can turn brief periods of low mood into prolonged episodes of depression. It must also be remembered that the authors of the MBCT manual tailored the mindfulness exercises to non-depressed clients with a history of relapsing depression, teaching them to recognise and remain 'open' to difficult emotions without engaging in ruminative thinking.

However, the results from this study suggest that MBCT may be of benefit to a wider range of patients than non-depressed patients with a history of relapsing depression. At the same time it is also plausible that some patients may benefit more from a course that placed less of an emphasis on mindfulness training (at least initially) and more of an emphasis on cognitive behavioural strategies (problem solving, assertiveness training, challenging maladaptive beliefs, etc.). Further research in the form of randomised controlled trials comparing group CBT to group MBCT would help address these important issues.

### Implications of this study and relationship to other work

A number of questions remain unanswered.

1) For individuals with affective disorders how does MBCT compare with group or individual CBT in terms of efficacy, acceptability and cost-effectiveness? A randomised controlled trial using both qualitative and quantitative research methods would be especially helpful in determining which elements of both mindfulness training and CBT clients use as antidotes to emotional distress.

2) When is the best time to introduce mindfulness training for patients with depression and anxiety? Should brief mindfulness training be introduced to patients with severe depression/anxiety right from the start of therapy or is it more effective for these patients to engage in individual/group cognitive behavioural therapy, deferring mindfulness training until some improvement in their mood?

3) Does long-term training in mindfulness meditation confer additional benefits for mental health compared with CBT?

### Strengths and limitations of the study

A strength of the present study was that it was conducted in a routine primary care setting, involving patients from a range of socio-economic backgrounds. Most of the participants had not practiced mindfulness meditation previously, and did not have fixed ideas about what to expect. The use of mixed methods in the evaluation of the study was also a strength.

However, there are several limitations to this exploratory study. This was a small study with no control group. Because there was no control group reductions in mean depression and anxiety scores cannot be directly attributed to the intervention. Only two quantitative measures were used and data was collected at only 2 points, one at the beginning of the group and the second three months after completing the course. The follow up duration was relatively short so it is not clear whether mindfulness training produces long term changes in affect. The author who led the group (AF) had no previous experience of running MBCT groups and a more experienced facilitator may have achieved better results. However, it should be noted that the participants felt that the facilitator was very empathic and understanding, and it was clear that a good therapeutic relationship developed between the facilitator and the group members.

## Conclusion

Mindfulness Based Cognitive Therapy, originally developed for non-depressed patients with a history of relapsing depression, may be acceptable and beneficial to patients with active depression and anxiety. Depression and anxiety are amongst the commonest problems seen by primary care professionals and group based interventions have the potential to offer cost-effective treatment to larger numbers of patients than individual therapy, alleviating the existing pressure on psychology services. However, if group based mindfulness approaches to mental health are to play a role in primary care then careful attention must be paid to training, capacity building and quality assurance. Further research is warranted that compares group based MBCT to other group based psychological interventions.

## Competing interests

The author(s) declare that they have no competing interests.

## Authors' contributions

SWM supervised all aspects of the study and analysis and carried out the qualitative interviews. He also carried out the statistical analysis and assisted in writing the paper.

AF ran the MBCT course and analysed the qualitative data, and wrote the initial draft of the paper, as well as contributing to the development of the final version.

## Pre-publication history

The pre-publication history for this paper can be accessed here:


